# Effects of biochar and crop straws on the bioavailability of cadmium in contaminated soil

**DOI:** 10.1038/s41598-020-65631-8

**Published:** 2020-06-12

**Authors:** Xuan Chen, Hong-Zhi He, Gui-Kui Chen, Hua-Shou Li

**Affiliations:** 1 Key Laboratory of Tropical Agro-Environment, Ministry of Agriculture/South China Agricultural University, Guangzhou, 510642 PR China; 2 Guangdong Engineering Research Center for Modern Eco-agriculture and Circular Agriculture/Key Laboratory of Agroecology and Rural Environment of Guangzhou, Regular Higher Education Institutions, Guangzhou, 510642 PR China

**Keywords:** Ecology, Plant sciences, Ecology, Environmental sciences

## Abstract

Numerous studies have been investigated the potential of biochar (BC) derived from various materials and crop straw (CS) to decrease the bioavailability of heavy metals in soil contaminated with cadmium (Cd), and thereby reduce their potential risk to human health and the ecological environment. However, little attention has been given to the comparison of heavy metal remediation efficiency using BC and CS such as peanut vine (PV) and rice straw (RS), especially in soil contaminated with Cd. Here, we explore if Cd bioavailability is affected in contaminated soil by BC and CS. Peanuts were grown in plastic pots, which contained BC or CS at 5% (dry weight, w/w) in controlled environment mesocosms. The bioavailability of Cd in contaminated soil was measured by Cd concentration in the plant and the concentrations of various forms of Cd in the soil. At the same plant age, growth with BC (compared with PV and RS) led to 13.56% and 8.28% lower rates of Cd content in the aboveground parts, 40.65% and 35.67% lower rates of Cd content in the seeds, yet 9.08% and 7.09% lower rates of Cd content in the roots, yet 35.80% and 28.48% lower rates of exchangeable Cd content in the soil. Moreover, BC amendment enhanced the biomass of peanut and physiological quality. Thus, BC had a greater impact on immobilizing Cd in the soil. The results imply that BC was more significantly (*P* < 0.05) remarkable in decreasing the Cd bioavailability and improving the biomass of peanut. BC has greater potential for enhancing soil quality and promoting peanut growth. In conclusion, this research demonstrates an understanding of employing BC as a promising inexpensive and eco-friendly amendment to remediate soil contaminated with Cd.

## Introduction

Nowadays, due to the expansion of industrial production and the rapid development of urbanization, the ever-increasing use of sewage irrigation, chemical fertilizers and pesticides in agricultural production result in the accumulation of heavy metals in the soil^1–3^. Cd is a carcinogenic heavy metal^4,5^, it has become one of the major sources of heavy metal pollution in soils and foods^6–8^. In the Toxic Substance and Disease Registry’s (ATSDR) priority list of hazardous substances, it ranks seventh. And studies have reported the toxic effects of Cd on biological systems^9,10^. In addition, Cd is extremely toxic to plants^11^. Compared to other heavy metals, the toxicity of Cd is 2 to 20 times higher. Some studies have shown that Cd is the fourth most toxic metal to vascular plants^12,13^. For plants, Cd is an unnecessary nutrient element, but plant roots can rapidly absorb it through nonspecific ion channels and via divalent metal transport (DMT)^14^, and when the Cd content of leaves exceed 5–10 μg·g^−1^, it becomes toxic^15^. Multiple studies have reported that excessive Cd can inhibit the normal metabolism of plants, which seriously affects the morphology and physiological processes of plants^16^. In general, Cd is absorbed by the roots, moved through via shoots and leaves, and then accumulated in grains and/or seeds^17,18^. Physiologically, Cd could lead to excessive production of reactive oxygen species (ROS), resulting in oxidative damage to plants, which may change plant morphological characteristics and cause significant reduction in yield^19^. In addition, Cd could interfere with chloroplast metabolic function. Studies about cell ultrastructure of plants under Cd stress have shown that chloroplast has drastic changes, especially the decomposition of thylakoid membranes and an increase starch^20,21^, which may be attributed to interference with chloroplast metabolism. Even worse, Cd could enter the human body system through food chain to affect human health^22^. When the accumulation of Cd reaches a certain amount, it would cause serious damage to the human kidney function and bones, and even lead to the occurrence of various cancers^23^. At present, scholars have studied various methods to reduce the accumulation of Cd in crops.

Recently, studies have paid considerable attention to soil amendments such as BC and CS that immobilize contaminants whilst promoting plant growth, and these amendments have been widely applied for decreasing the bioavailability of heavy metals in soils^23,24^. BC is a carbon-rich solid material made by pyrolysis of biomass under oxygen limited conditions^25^. Typically, the physical and chemical properties of biochar what make it environmentally friendly^25^, BC has a high porosity and large specific surface area, and strong ion exchange capacity, which offer a great potential for application to soils for carbon sequestration, greenhouse gas emission reduction, soil fertility improvement, and contaminated soil remediation^26–28^. BC is used as a soil conditioner to improve soil fertility as well as to adjust the soil pH, moisture, air, and temperature conditions^24^. Previous researches have reported the biochemical effects of BC on heavy metal adsorption in soil and water in recent years^29–31^. Cui *et al*.^32^ reported that wheat straw BC reduced the Cd bioavailability up to ~90% as compared to control (2.5 mg Cd/kg) and exhibited stable property based on the long-term incubation. Bashir *et al*.^33^ showed that biochar significantly reduced the bioavailability of Cd and Cr by 85% and 63%, respectively. And when the application amount of biochar was 15 g·kg^−1^, the extractable Cd in Cd-contaminated and Cr-Cd-contaminated soil could be reduced by 29% and 32%, respectively. Other studies presented that sugarcane straw derived BC could reduce the Zn, Cd, and Pb effectively in the pore water within acidified soil, thereby reducing their mobility and indicating that the main mechanism was the metal adsorption on BC^34^. In a similar way, the application of CS as a fixative in soil contaminated with heavy metals may play an effective role for metal bioavailability. CS, as the main by-product of crops, not only has a positive effect on the soil structure, but also can loosen the soil, thus promoting the growth and development of crops^35^. Moreover, many studies have demonstrated that straw returned to the field could produce dissolved organic carbon (DOC) which acted as an organic ligand to adsorb heavy metals in the soil^36^. The organic ligand in soil solution could affect the formation of heavy metal complexes^37^.

In this work, peanuts were used as experimental materials. Peanut (Arachis hypogaea) is one of the most important oilseed crops in the world and it has a strong absorption capacity for Cd^38^. Some studies have shown that even on soils with relatively low total or available Cd concentrations (<0.5 mg·kg^−1^), the Cd in seeds was higher than the maximum allowable concentration (MPC, 0.05 mg Cd/kg). In addition, the Cd concentrations at the top of plant always exceeded that in kernel or seeds. Furthermore, the testa in peanut kernel contained 50 times more Cd than the embryonic axis and cotyledons^39^. Wang *et al*.^40^ reported the Cd content in peanut kernel in Yantai ranged from 0.027 to 0.280 mg·kg^−1^, with an average of 0.1048 mg·kg^−1^, which surpassed hygienic quality standard of green peanut kernel issued by Ministry of Agriculture (HQSGPK). The rate beyond the HQSGPK reached 37.5% among all tested fields. So the development of peanut lines with food safety (Cd ≤0.2 mg/kg, GB 2762-2012); Cd ≤ 0.1 mg/kg for the CODEX STAN 193-2015) would represent a major advance for the peanut industry. Our research goal is to develop an efficiency way for the immobilization of heavy metals in contaminated soil to assist the peanut safety.

Here, we examine how plants respond to Cd stress by measuring the biomass of peanut and physiological quality, and Cd content in different parts of peanut and exchangeable Cd content in soil. We performed a small scale experiment with peanut grown under BC addition and CS addition. The bioavailabilities of Cd in contaminated soil under BC addition and CS addition were analysed by laboratory simulation experiment. Results showed that BC and CS are good candidates for the remediation of soils contaminated with heavy metals. The efficiency of the BC and CS for the immobilization of heavy metals in contaminated soil should be carefully evaluated for each specific site prior to large-scale application. Therefore, the specific objectives of this paper were to, (1) determine the effect of BC and CS application on the bioavailability of Cd in contaminated soil, (2) compare the efficiency of BC and CS for remediation of soil contaminated with Cd, and (3) measure the effects of BC and CS application on plant yield and physiological response. This study aims to provide fundamental insights into the feasibility of using BC and CS to decrease the Cd bioavailability in contaminated paddy soil.

## Materials and methods

### Experiment materials

The topsoil (0–20 cm) was collected from an abandoned paddy field in Tianhe District, Guangzhou City, Guangdong Province, China, and it was classified as latosolic red soil according to the Chinese soil classification^41^. Soils were air-dried, crushed and sieved through the sieve of 2 mm meshes. The soil pH was 5.5, the organic matter content was 12.13 mg·kg^−1^, and the total N, P and K concentrations of soil were 317.60 mg·kg^−1^, 292.68 mg·kg^−1^, 2236.61 mg·kg^−1^, respectively, and the available N, P and K concentrations of soil were 27.13 mg·kg^−1^, 1.14 mg·kg^−1^, 64.14 mg·kg^−1^, respectively. The total Cd content of the soils was below the detection limit of a graphite furnace (Z700P, Jena, Germany).

The peanut cultivar selected in this experiment was Yueyou No. 7 of Guangdong. The selected peanut shell BC was purchased from Sanli Company of Shangqiu, Henan Province, China. The total nitrogen and carbon content of BC were 7.20 g·kg^−1^ and 521.31 g·kg^−1^, respectively. PV and RS were collected from a farm in the Ecology Department of South China Agricultural University, their total nitrogen and carbon contents were 7.61 g·kg^−1^, 501.77 g·kg^−1^ and 6.36 g·kg^−1^, 437.82 g·kg^−1^, respectively. The total Cd contents of BC and CS were below the detection limit of graphite furnace atomic absorption spectrometry (Z700P, Jena, Germany). All three carbon-based materials were crushed and sieved through the sieve of 2-mm meshes and they were mixed well into the experimental soil.

### Sampling and analysis

#### Soil and materials characterization

The physicochemical properties of soil were measured using the methods described by Bao^42^. Briefly, the soil pH was determined by a soil/water slurry ratio of 1:2.5 (w/v). The determination of soil organic matter was based on the method using potassium dichromate (K_2_Cr_2_O_7_) and concentrated sulfuric acid (H_2_SO_4_) to oxidize the soil carbon before titrating with ferrous sulfate (FeSO_4_). The total and available contents of phosphorus, potassium and nitrogen of the experimental soil were determined according to Bao^42,43^. The total Cd content of the soil was determined using a Z700P atomic absorption spectrometer (Z700P, Jena, Germany). The concentrations of various forms of Cd in soil were analyzed using a sequential extraction procedure developed by Tessier *et al*.^44^.

The carbon contents of BC and CS were evaluated via C/N analyzer (TOC MULTI N/C 2000, Analytik Jena), and their nitrogen contents were determined according to Li *et al*.^45,46^.

#### Plant analysis

Each plant sample was divided into four parts, including the shoot, shell, seed and root. The number of peanut pods and seeds in each treatment were counted for statistic analysis, and the dry matter weight was determined after the samples were oven-dried at 105 °C for 0.5 h and then oven-dried at 75 °C until their weight was constant.

Peanut leaves were collected and cut into pieces, then immersed in a mixture of ethanol and acetone (1:1; v/v) for measuring the concentrations of chlorophyll and proline by ultraviolet spectrophotometer. The concentrations of soluble protein, soluble sugars, and crude fat in peanut were measured using Commassie brilliant blue G-250 staining, anthrone colorimetry and Soxhlet extraction, respectively. All methods described above followed Modern Plant Physiology Experimental Guidelines^45^. The Cd concentration in the plant samples was extracted using microwave digestion and determined using an atomic absorption spectrometer (Z700P, Jena, Germany).

### Experiment design

The experiment was carried out on a farm (23°16′N, 113°37′E) of Ecology Department of South China Agricultural University in Tianhe District, Guangzhou City, Guangdong Province, China, under natural sunlight. To prepare a Cd-contaminated soil, 3 L solution containing 48 mg of CdCl_2_ in deionized water was sprayed onto 3 kg soil, then the soil was gradually diluted with 45 kg of clean soil until the final concentration of Cd^2+^ reached 1 mg·kg^−1^. Subsequently, a total of 4 kg of contaminated soil spiked with Cd was placed into a plastic pot (diameter 20 cm, height 16 cm). Peanut shell BC, PV, and RS were added into the pots at a rate of 5% (dry weight, w/w) and mixed thoroughly to ensure uniformity. This experiment consisted of 4 treatments and every treatment had 3 replications. The soil contaminated with Cd without any amendment was set as the control. To guarantee the regular growth of plants, each pot was supplemented with urea, Ca(H_2_PO_4_)_2_, and KCl at a dose of 0.2 g·kg^−1^, 0.4 g·kg^−1^, and 0.3 g·kg^−1^, and these pots were arranged in a randomized complete block design. The pots were irrigated with deionized water to 70% of the field water holding capacity, and allowed to equilibrate for 4 weeks. Peanut seeds of uniform size were sowed in a sandy bed. At the four-leaf stage, uniform seedlings were selected and transplanted into the plastic pots, 3 seedlings were kept in each pot. Regular watering (2 to 4 times a week) with deionized water was maintained to prevent drought stress to the plants. After 4 months (from March 8 to July 8, 2016) the mature peanut were harvested, the soil and the plant were sampled, ground and sieved for further analysis.

### Statistical analysis

The data was analyzed via Microsoft Office Excel 2007 and Data Processing System (DPS 7.05), and the data of the study was presented as mean value and standard deviation. Significant differences were tested among treatments by one-way ANOVA and via post hoc least significant difference tests (LSD) for multiple comparisons at a 5% significance level. All statistical analyses were carried out using SPSS 17.0 and the data was graphed using Origin 8.0.

## Results

### Soil pH

**Figure 1:**
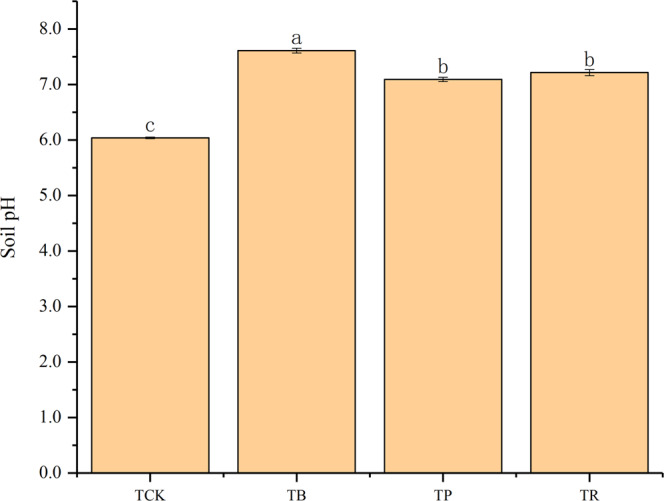
Effect of biochar and crop straw application on soil pH. Treatments: T_CK_: control, T_B_: biochar addition, T_p_: peanut straw addition, T_R_: rice straw addition. Error bars indicate standard error of the means (n = 3). Different letters indicates significant difference among treatments (P < 0.05).

BC and CS had strong effects on soil pH. Particularly in the BC treatment. Relative to the control, both BC and CS application could effectively increase the pH of soil (Fig. 1). The pH of soil was 24.00% higher at *T*_*B*_. Meanwhile, CS increased pH by 17.54% (*T*_*R*_) and 15.54% (*T*_*P*_), respectively (*P* < 0.05), whereas there was non-significant difference between *T*_*R*_ and *T*_*P*_ treatments.

### Infrared spectra of DOM in BC and CS

**Figure 2:**
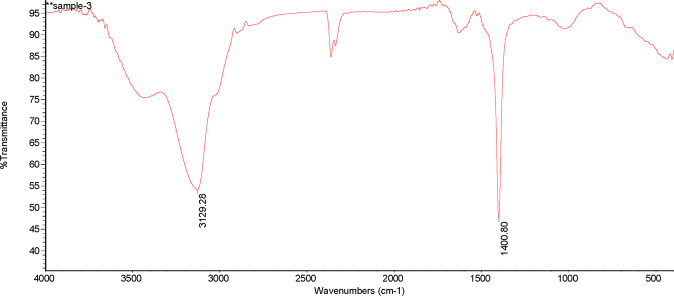
Infrared spectra of DOM in biochar.

It can be seen from Table 1 and Fig. 2 that there were two obvious absorption peaks in the DOM of BC at 1400.80 cm^−1^ and 3129.28 cm^−1^. The absorption peak at 1400.80 cm^−1^ showed that there were more CH_3_ and CH_2_ in the aliphatic group and fewer C = C and amido bonds on the aromatic group. A wide absorption band at 3129.28 cm^−1^ indicated that the contents of phenols, –COOH, and alcohols were much higher.

**Table 1:** Assignment of characteristic absorption bands in infrared spectra.

Absorption band position/cm^−1^	Absorption band assignment
650-520	Stretching vibration of -OH (carbohydrates)
870	Carbonate substance
1020-970	Stretching vibration of C-O or stretching vibration of inorganic SiO (carbohydrates)
1080–1020	Asymmetric stretching vibration of C-O (phenols or alcohols)
1170–1150	Stretching vibrations of C-OH and C-O (aliphatic)
1220–1210	Asymmetric stretching vibration of C-O or deformable vibration of N-H (hydroxyl)
1250–1230	Stretching vibration of C-O or stretching vibration of SiO in organosilicon compounds (phenols)
1460–1400	Symmetric deformable vibrations of -CH_3_ and -CH_2_, and asymmetric stretching vibration on hydroxyl group, or stretching vibration of C-OH (aliphatic)
1555–1540	Deformable vibration of -N-H (secondary amide)
1650–1600	Stretching vibration of -C = O, stretching vibration of C = C on aromatic group or antisymmetric vibration of organic carboxylate COO- (aldehyde, ketone)
1720–1690	Stretching vibration of -C = O, stretching vibration of C = O in hydroxyl group (hydrogen bond formed between molecules and within molecules)
2870–2850	Symmetric stretching vibrations of -CH_3_ and -CH_2_
2900	Stretching vibration of C-H (aliphatic)
2930	Asymmetric stretching vibration of -CH_2_ (aliphatic)
2950	Asymmetric stretching vibration of -CH_3_ (aliphatic)
2060–3030	Stretching vibration of -C-H (aromatic nucleus)
3500–3300	Stretching vibrations of -COOH and -OH or stretching vibration of N-H and hydrogen bond association

According to Huang (2013), etc.

**Figure 3:**
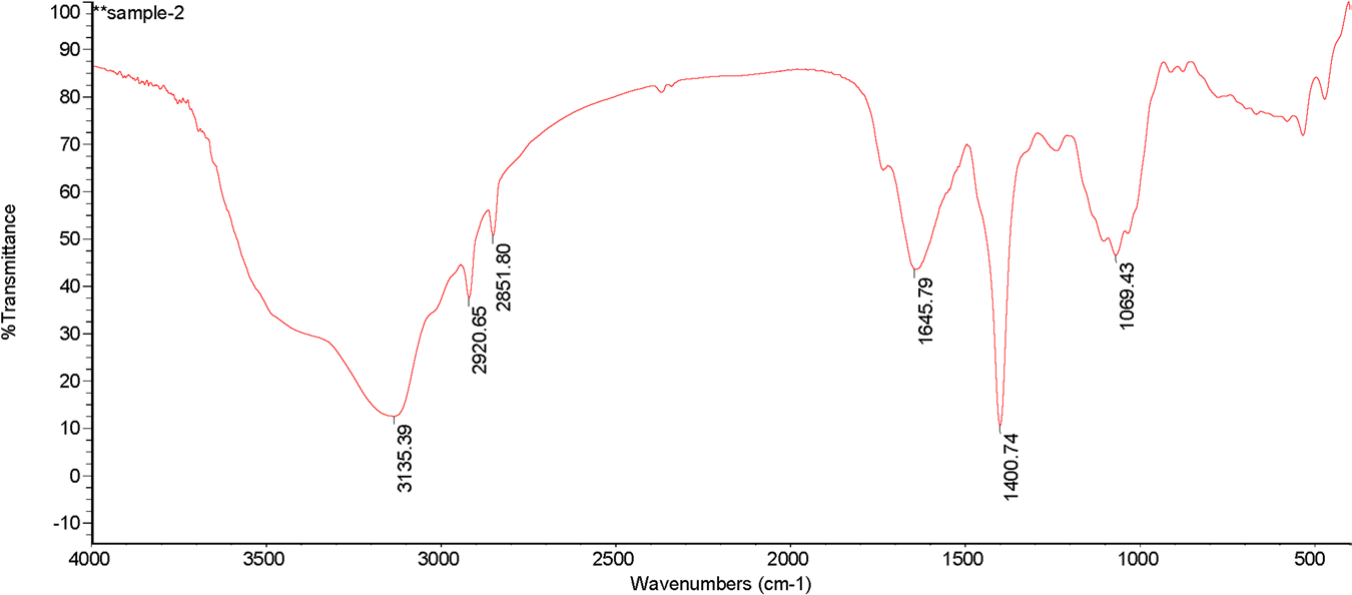
Infrared spectra of DOM in peanut vine.

**Figure 4:**
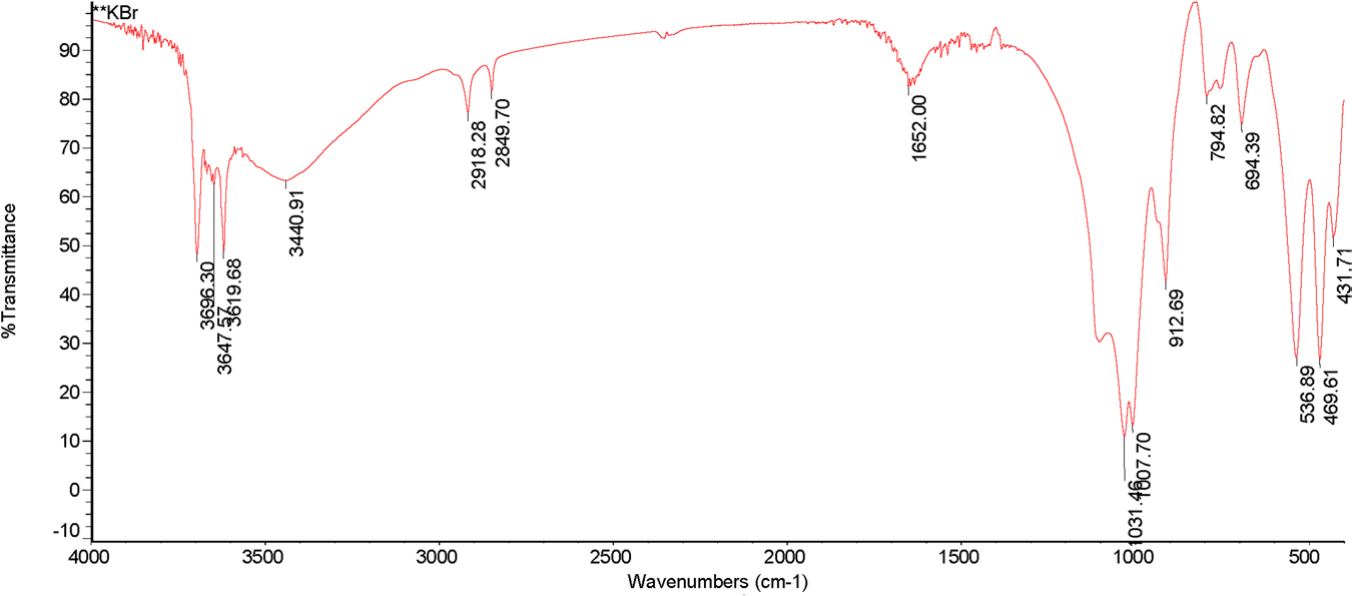
Infrared spectra of DOM in the decomposition products of peanut vine.

From Figs. 3 and 4, it presented that in the DOM of PV, there were four obvious absorption peaks at 1069.43 cm^−1^, 1400.74 cm^−1^,1645.79 cm^−1^ and 3135.39 cm^−1^. The absorption peak at 1069.43 cm^−1^ indicated that there were more C-O on the phenols or the alcohols. Besides, there were more CH_3_ and CH_2_ in the aliphatic group at 1400.74 cm^−1^ and fewer amido bonds. Moreover, the absorption peak at 1645.79 cm^−1^ showed that the contents of aldehyde, ketone and aromatic group were much higher. In addition, the absorption peak was widened at 3135.39 cm^−1^, and the contents of phenols and alcohols increased. Relative to PV, a total of six obvious absorption peaks appeared in the DOM of the decomposition products of PV. At 469.61 cm^−1^ and 536.89 cm^−1^, the carbohydrate content was much higher. Also, the degree of absorption at 1000 cm^−1^ was relatively weak, which means that the C-O of phenols or alcohols was more converted to carbohydrates. Plus, the absorption peaks were widened at 3440.91 cm^−1^ and the two small absorption peaks at 3619.68 cm^−1^ and 3696.30 cm^−1^ indicated that an increase in the contents of -COOH, phenols and alcohols.

**Figure 5:**
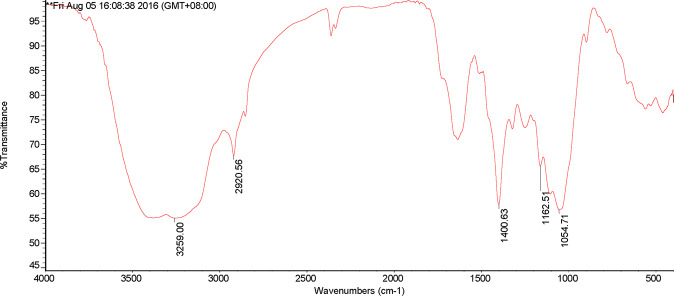
Infrared spectra of DOM in rice straw.

**Figure 6:**
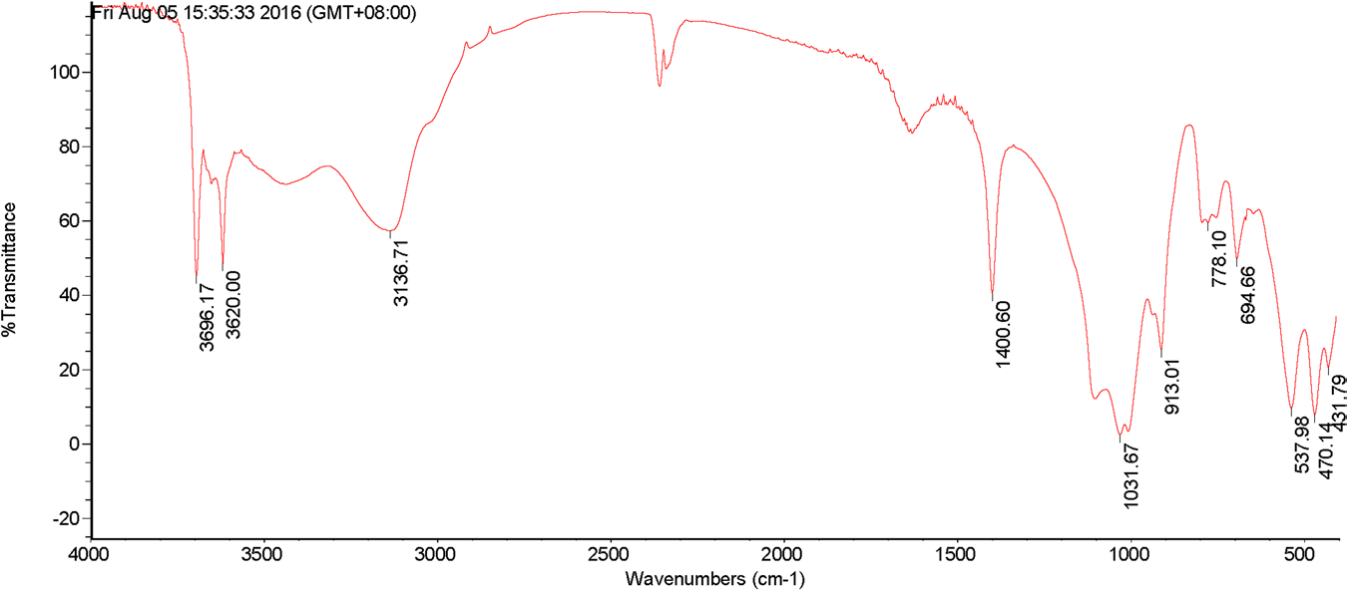
Infrared spectra of DOM in the decomposition products of rice straw.

Furthermore, from Figs. 5 and 6, there were three obvious absorption peaks in the DOM of RS at 1054.71 cm^−1^, 1400.63 cm^−1^ and 3259.00 cm^−1^, and seven distinct absorption peaks in the DOM of the decomposition products of RS, including 470.14 cm^−1^, 537.98 cm^−1^, 1000 cm^−1^, 1400.60 cm^−1^, 3136.71 cm^−1^, 3620.00 cm^−1^, and 3696.17 cm^−1^. The results were consistent with the DOMs of PV and the decomposition products of PV, respectively.

### The contents of total Cd and various forms of Cd in soil

**Figure 7:**
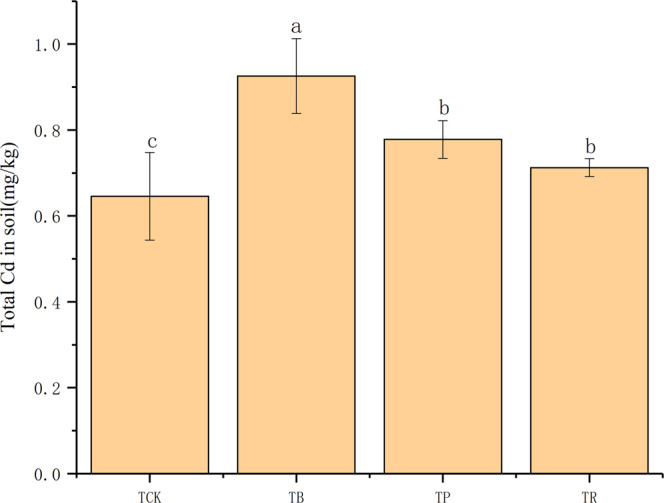
Effect of biochar and crop straw application on soil total Cd content. Treatments T_CK_: control, T_B_: biochar addition. T_P_: peanut straw addition, T_R_: rice straw addition. Error bars indicates standard error of the means (n = 3). Different letters indicate significant difference among treatments (*P* < 0.05).

**Figure 8:**
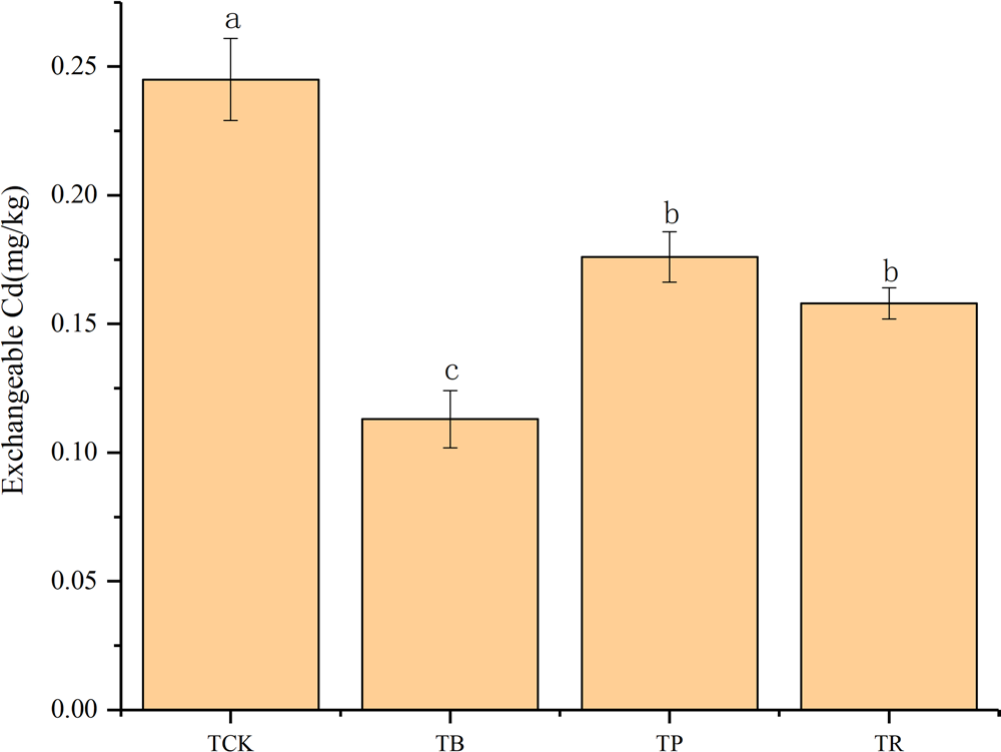
Effect of biochar and crop straw application on soil exchangeable Cd content. Treatments: T_CK_: control, T_B_: biochar addition, T_P_: pearnut straw addition, T_R_: rice straw addition. Error bars indicate strandard error of the means (n = 3). Different letteres indicate significant difference among treatments (*P* < 0.05).

**Figure 9:**
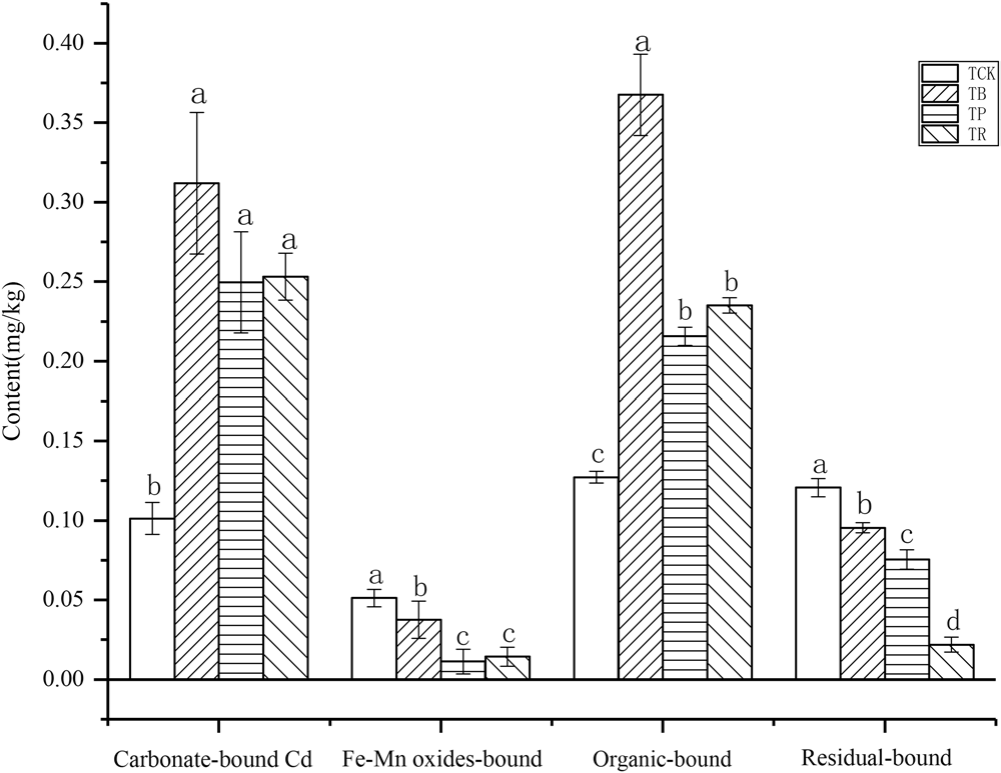
Effect of biochar and crop straw application on the contents of various forms of Cd in soil. Treatments: T_CK_: control, T_B_: biochar addition, T_P_: peanut straw addition, T_R_: rice straw addition. Error bars indicates standard error of the means (n = 3). Different letters indicate significant difference among treatments (*P* < 0.05).

As expected, in both BC and CS, the total Cd contents in soil were all consistently higher than *T*_*CK*_ (Fig. 7) and the exchangeable Cd contents were lower than *T*_*CK*_ (Fig. 8). In this study, growth under BC addition (compared with PV addition and RS addition) led to 18.97% and 29.90% higher rates of total Cd content in the soil, yet 35.80% and 28.48% lower rates of exchangeable Cd content in the soil (*P* < 0.05). Furthermore, BC and CS could effectively increase the contents of carbonate-bound Cd and organic-bound Cd in soil (Fig. 9). Particularly in the biochar treatment, the highest increase reached to 48.07% and 52.94%, respectively.

### Cd accumulation in the tissues of peanut

**Figure 10:**
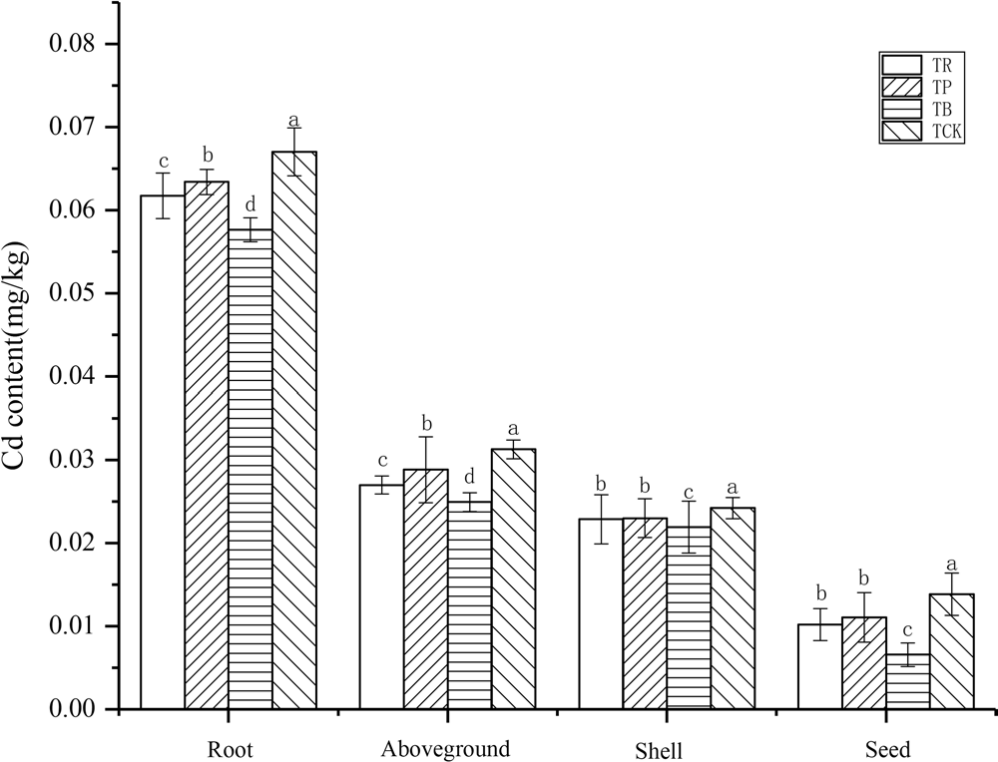
Effect of biochar and crop straw addition on Cd accumulation in the tissues of peanut. Treatments: T_CK_: control, T_B_: biochar addition, T_P_: peanut straw addition, T_R_: rice straw addition. Error bars indicate strandard error of the means (n = 3). Different letters indicate significant difference among treatments (*P* < 0.05).

The Cd concentrations in the tissues of peanut were given in Fig. 10. When compared at the same age, the concentrations of Cd in the different parts of the peanut followed the order: root > aboveground > shell > seed. When compared at the same tissue, the concentrations of Cd under different materials followed the order: root > aboveground > shell > seed. Compared to the control, with either *T*_*B*_ or *T*_*R*_ or *T*_*P*_, the Cd concentrations in roots decreased by 13.99%, 7.89% and 5.40%, respectively (*P* < 0.05), and led to 20.30%, 13.70% and 7.80% lower rates of Cd concentrations in aboveground parts, yet 9.54%, 5.52% and 5.03% lower rates of Cd concentrations in shells, yet 28.77%, 5.84% and 5.30% lower rates of Cd concentrations in seeds. In addition, there was non-significant difference between *T*_*R*_ and *T*_*P*_ treatments.

### Physiological parameters, biomass and yield of peanut

**Figure 11:**
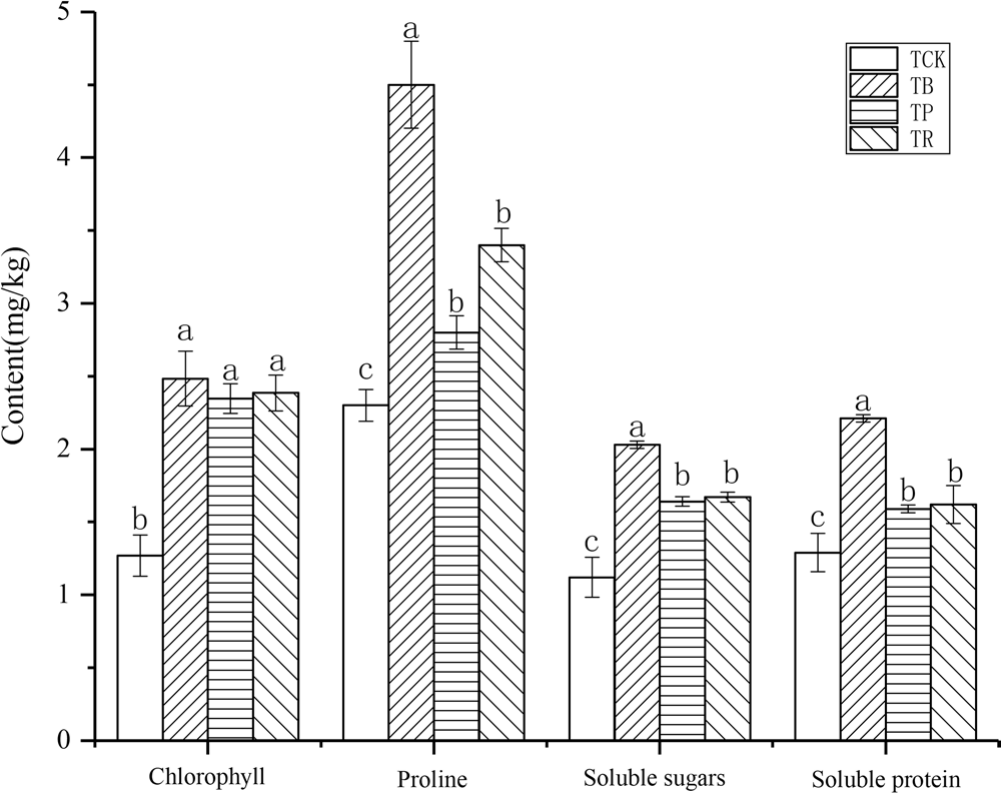
Effect of biochar and crop straw application on physiological parameters in peanut. Treatments: T_CK_: control, T_B_: biocchar addition, T_P_: peanut straw addition, T_R_: rice straw addition. Error bars indicate standard error of the mean (n = 3). Different letters indicate significant difference among treatments (*P* < 0.05).

**Figure 12:**
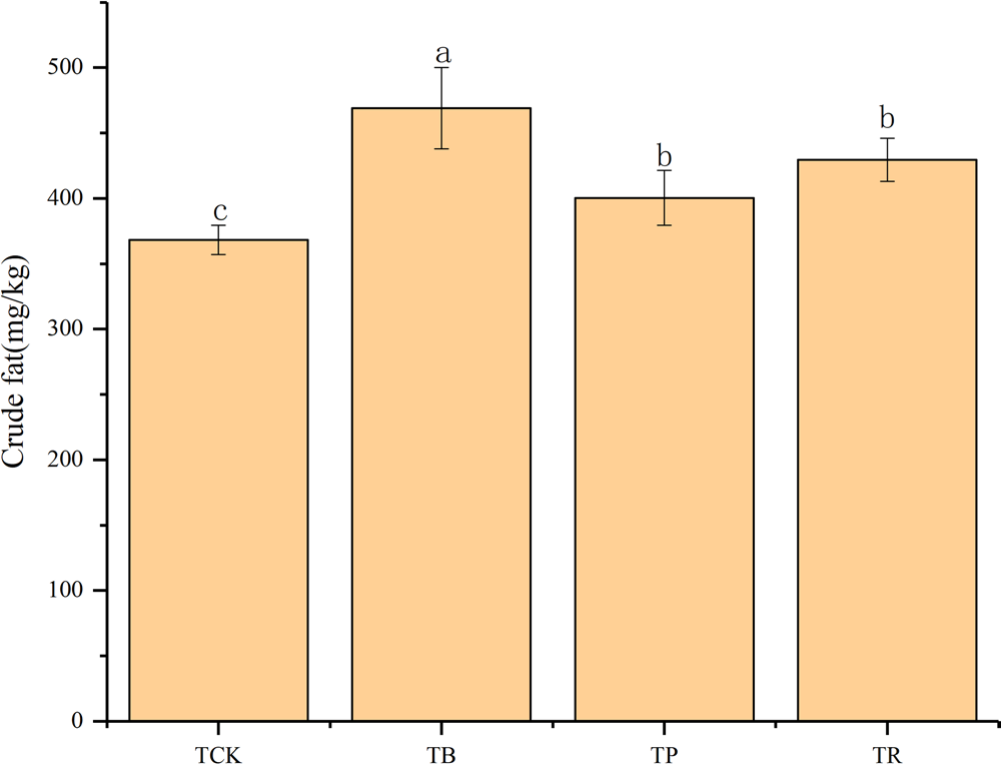
Effect of biochar and crop straw addition on crude fat in peanut. Treatments: T_CK_: control, T_B_: biochar addition, T_P_: peanut straw addition, T_R_: rice straw addition. Error bars indicates strandard error of the means (n = 3). Different letters indicate significant difference among treatments (*P* < 0.05).

Both BC and CS had clear effects on many physiological parameters (Figs. 11 and 12). Particularly in the BC treatment. Relative to CS, BC had an approx. 95.61% higher chlorophyll, 95.65% higher proline, 81.25% higher soluble sugars, 71.32% higher soluble proline, and a 27.37% higher crude fat, as compared with the control. On the other hand, BC had 32.35% and 60.71% higher proline, yet 21.56% and 23.78% higher soluble sugars, yet 36.42% and 38.99% higher soluble proline, yet 9.20% and 17.13% higher crude fat compared with RS and PV. Nevertheless, all physiological parameters did not differ significantly between the two CS (*P* > 0.05).

Table 2 presents the biomass and yield of peanut with the applications of BC and CS. The applications of BC and CS significantly increased the biomass and yield of peanut. Particularly in the BC treatment, the highest decrease of biomass in aboveground parts and underground parts reached to 86.49%, and 75.47%, respectively(*P* < 0.05), and the highest decrease of yield in seeds per plant reached to 61.42% (*P* < 0.05).

**Table 2:** Effect of biochar and crop straw addition on the biomass and yield of peanut.

Treatments	Biomass				Yield	
	Aboveground (g·plant^−1^)	Underground (g·plant^−1^)			Number of effective pods per plant	Number of seeds per plant
		Roots	Seeds	Shells		
T_CK_	9.45 ± 1.54c	1.61 ± 0.29c	6.26 ± 0.46c	3.75 ± 0.34b	15.00 ± 0.58c	19.00 ± 1.15c
T_B_	17.61 ± 2.33a	4.05 ± 0.09a	11.17 ± 0.55a	5.17 ± 0.32a	21.00 ± 0.57a	30.67 ± 0.58a
T_P_	14.00 ± 1.38b	2.16 ± 0.09b	9.91 ± 1.62b	4.62 ± 1.14b	15.33 ± 1.53b	21.00 ± 1.53b
T_R_	14.97 ± 1.25b	2.37 ± 0.24b	10.50 ± 0.82b	4.85 ± 0.77b	16.33 ± 0.57b	22.00 ± 1.15b

Treatments: TCK: control, TB: biochar addition, TP: peanut straw addition, TR: rice straw addition.

All values are presented as mean ± standard error (n = 3), different letters in the same row indicate significant differences between treatments (P < 0.05).

## Discussion

### Biochar had a greater impact on immobilizing Cd in the soil

This work shows that the contents of total Cd and exchangeable Cd in the soil were affected by biochar and crop straw, with a higher total Cd and a lower exchangeable Cd in the biochar and crop straw treatments mainly due to the increase in pH. One of the mechanisms for the pH increase after the application of these two materials is probably due to the considerable amount of ash and base cations^47–49^. On the one hand, for biochar, the basic cations existing on the feedstock biomass could be converted into oxides, hydroxides and carbonates produced in the pyrolysis process of biochar, which may contribute to the increase of soil pH^50^. On the other hand, the high alkalinity of these two materials^25^, and the subsequent release of base cations, especially Ca^2+^ and K^+^, and the replacement of soil exchangeable Al^3+^ and H^+^ by these cations on the soil’s negatively charged sites could greatly increase soil pH^51^. In addition, the decarboxylation of organic anions induced by these two materials, owing to increasing attack of organic anions by microbes, which may consume H^+^ from the soil solution^52,53^.

The response of exchangeable Cd content to pH is important for understanding the effect of organic materials on the bioavailability of Cd. The increase of the pH resulted in the hydrolysis of heavy metal cations to form oxide precipitations, which decreased the content of exchangeable Cd in soil^54^. In addition, many studies have shown that the application of organic materials (including crop straw and biochar) can significantly affect the adsorption and desorption behavior of heavy metal in soil^55,56^. The most direct effect of biochar and crop straw was to bring a large amount of dissolved organic matter (DOM) into soil. DOM is the most active component of organic matter and has active groups. It is easy to chelate and complex with Cd as ligands, and then change the availability of Cd^57^.

The negative charge on the surface of clay minerals, hydrated oxides and organic matter in soil increases with the increase of soil pH, which increases the adsorption of Cd^2+^, which promotes the formation of CdCO_3_ and Cd(OH)_2_ precipitates^58^, which may be the reason for the increase of the content of carbonate-bound Cd in soil^59^, and the reason for the increasing the content of organic-bound Cd in the soil might be due to the abundant carboxyl groups in biochar and the decomposed products of crop straw contained more carboxyl groups, aldehyde groups, ketone groups, and aromatic substances, so the retention ability of ions was enhanced, and a significant colloidal characteristic was gradually exhibited^60^. Furthermore, biochar and crop straw can reduce the toxicity of Cd to peanuts by increasing the concentration of total Cd in the soil. In our study, we found that these two materials can significantly increase the concentration of total Cd. The reason might be due to the numerous organic functional groups in the biochar, such as –C-OH, –C=O and COO–, which could complex heavy metal ions^61^. As can be seen from Fig. 3, the effects of the three carbon-based materials on the total Cd were consistent, the organic matter produced by the decomposition of crop straw also played the same role^62,63^.

In summary, the inhibition effect of biochar and crop straw on Cd adsorption is mainly through chemisorption. The inhibition effect of biochar mainly relies on the ion exchange of metal cations on its surface^64^ and the precipitation or complexation of metal ions on biochar with minerals or functional groups^54^. The inhibition effect of crop straw mainly relies on the decomposition products produced by soil microbes, and there are plenty of functional groups on the surface of crop straw, which can complexation metal ions^65^.

### Biochar has a positive effect on reducing Cd content in peanut

The results of this study show that the addition of biochar and crop straw could effectively reduce the absorption of Cd in peanut. Some studies presented that the addition of biochar could inhibit the absorption of Cd in rapeseed^66^ and other plants, such as rice^67^, wheat^68^, spinach^69^. In this research, the reason that biochar could reduce the Cd concentration in different parts of peanut might be that on the one hand, biochar could increase Cd concentration bound to the soil organic matter. On the other hand, it might be due to the reduction in pore water Cd concentration^70^. For crop straw, studies have shown that the addition of crop straw not only increased the pH of soil^71^, but also improved the metal adsorption by ligands in organic matter^63,72,73^, so as to reduce the absorption of Cd by plants. Ok *et al*.^74^ found that the use of rapeseed residue amendment could decrease the easily accessible fraction of Cd by 5 to 14%, thus reducing the bioavailability of Cd. Xu *et al*.^72^ reported that after adding rice straw and wheat straw, the accumulation of Cd in maize shoots decreased by 69.5 and 66.9%, respectively. Zeng *et al*.^65^. found that in the cassava–peanut intercropping system, the uptake of Cd by the two crops was significantly reduced after the addition of crop straw. In this work, the results indicate that the most easily accumulated Cd parts in peanuts were roots, followed by shoots and pulses(grains). The higher concentration of Cd in the roots suggested that one of the ways to inhibit the transfer of Cd from roots to shoots might be by locating toxic metals in the plant tissues^75^.

### Biochar and crop straw additions enhanced the biomass of peanut and physiological quality

The present study indicates that these two materials increased the physiological quality of peanuts compared to the control. In this study, the increase in chlorophyll contents might be due to the reversal of Cd-induced toxicities in the plants with organic materials applications^68,76^. In addition, the increase in proline, soluble protein, soluble sugars, and crude fat with the additions of biochar and crop straw might be due to the increase in mineral nutrients and decrease in Cd concentration in the plants^77^.

Further, the biochar and crop straw exerted a positive effect on enhancing the biomass and yield of peanut. On the one hand, the biomass and yield of peanut are well known to be improved by the additions of biochar and crop straw, and this improvement is attributed to the modification of the physicochemical and biological characteristics of the cultivated soil^77,78^. On the other hand, the application of these two materials can reduce the bulk density and increase the total porosity of the soil, which could provide a good space for the root growth.

## Conclusions and perspectives

This study showed biochar and crop straw had significant effects on decreasing of Cd availability in soil and Cd concentration in peanut pulses, especially biochar, it had a greater impact on immobilizing Cd. At the same peanut plant age, growth with biochar (compared with peanut vine and rice straw) led to 13.56% and 8.28% lower rates of Cd content in the aboveground parts, 40.65% and 35.67% lower rates of Cd content in the seeds, yet 9.08% and 7.09% lower rates of Cd content in the roots, and 35.80% and 28.48% lower rates of exchangeable Cd content in the soil. In the meantime, Cd uptake by the peanut markedly decreased, and the biomass and yield of peanut markedly increased. These findings suggested that biochar could be used as an ecological friendly amendment, and served for mitigates Cd pollution in the soil-grain system.
